# Amiloidosis, a mysterious disease, still underestimated

**Published:** 2008-04-15

**Authors:** Penescu Mircea

**Affiliations:** * ”Carol Davila” University of Medicine and Pharmacy, Bucharest, Romania

**Keywords:** systemic amyloidosis, localized amyloidosis, AL amyloidosis, AA amyloidosis, diagnosis of amyloidosis

## Abstract

Amyloidosis, 150 years after being identified, still remains a mysterious disease, full of mystery, question marks and challenges, almost always ignored from the start, with terrible symptoms and terrible prognosis.

The mechanics of the formation, persistence and setting of the amyloid fibrils are still under discussion.

This article wishes to point out some of the main characteristics of this disease and the involved proteins by referring to the history, the pathogenesis, the histology, the diagnosis and a few aspects of the prognosis of this disease in the hope that future research will bring to light the answers for the mystery of amyloidosis along with more efficient therapies.

Amyloidosis is a vast pathology chapter, of which very little has been documented in Romanian specialty literature.

It is a strange disease and its extremely diverse palette of symptoms – from its asymptomatic presence in certain tissues to its systemic disease aspects – represent a very significant subject.

The aetiopathogeny of this disease, known for about 150 years, can not be explained entirely even today. Despite all the progress in different fields of science which lead the way to many diverse theories regarding the causes of amyloidosis, the origin of the disease is still only partially known.

The mechanics of the formation, persistence and setting of the amyloid fibrils are still under discussion.

It is certain that there is an over-production of precursor protein fibrils - with an altered chemical structure, as a result of a faulty protein mechanism – these precursors having a particular stereochemical configuration and an extreme resistance to any enzyme digestion.

In addition to over-production, the altered clearance of these molecules and abnormal processing, all represent a strong triggering mechanism.

Pathological fibril proteins originate in a transmissible genetic fault, either appearing as a metabolic irritative reaction to an intense inflammatory stress – which is not specific or is secondary to some well defined systemic diseases.

From a histopathological point of view – the disease has an extreme lesion polymorphism. 

The progress recorded in deciphering the amyloid structure has been done in 2 ways:

• Through microscopic studies on a variety of tissues

• Through “in vitro” studies on amyloid fibril proteins 

Studies have evolved from optical studies with classic colourants like Congo Red and Thioflavin to microscopic studies with polarized light, immunohistochemical studies spectrophotometry or electronic microscopy.

Clinically, amyloidosis can be considered as one of the most devastating diseases, as a proportion of the affected population (a major part still not being diagnosed) or as a suffering degree – (amyloidic nephropathy, Alzheimer’s disease, spongiform encephalopathies, type II diabetes mellitus)

All the intense and constant concern of some medical, biochemical, biophysical or genetical specialists still can not answer at least three problems:

• What are the mechanisms that determine the specific pattern of amyloid deposits location

• Why don’t all patients which have favourable conditions for Amyloidosis actually get Amyloidosis

• What is the explanation of the therapeutical resistance to numerous attempts to neutralise or dynamically reduce amyloid deposits

Amyloidosis, 150 years after identifying it, still remains a mysterious disease, full of mystery, question marks and challenges, almost always ignored from the start, with terrible symptoms and terrible prognosis.

## Definition:

Amyloidosis is a heterogeneous group of affections, usually of an unknown origin, with a fundamental mechanism of extra cellular deposits of autological proteins with a fibrilar ultrastructure and specific tinctorial properties. It is clinically characterized through polymorphic symptoms, in connection to the locality and quantity of deposited amyloid.

It is important to underline that the definition of amyloidosis is notably pathophysiological, which is the common element characterizing all different types of amyloid. All types of amyloid fibril have the following properties:

a. Secondary structure of folded beta pleated sheet; 

b. Green birefringence on polarised light microscope after Congo Red colouring;

c. Fibrilar quaternary structure with characteristic ultra structural aspect (pile of thorns).

## History:

1842 *Rokitanski* describes the lardaceous degeneration

1858 *Virchow* introduces the term amyloid and describes its colouring properties

1859 *Friedreich, Kekulé* define the protein structure of the A

1920 *Schmiederberg Schmiederberg* proves that the A.A structure of the amyloid is similar to that of the serum immunoglobulin

1922 *Bennhold* introduces the Congo Red colouring

1927 *Divry* describes the amyloid birefringence with the polarised light microscope

1931 *Magnus-Levy* determines the association between the amyloid and the existence of the Bence-Jones protein

1959 *Cohen and Calkins* prove with the electronic miscroscope the branchless fibril structure of the amyloid

1968 *Eanes and Glenner* demonstrate that the amyloid has a folded type beta sheet secondary structure by using X ray difraction

1971 *Glenner* analyses the primary structure of the multiple Myeloma amyloid

1972 *Ein,Glenner,* analyse the primary structure of the amyloid from chronic inflammatory diseases

1980 The biochemical classification of the disease is established

The suspected diagnosis of systemic amyloidosis can be rarely made based on a suggestive clinical picture. More often, the presence of a monoclonal gammapathy or a chronic inflammatory disease, leads to this diagnosis. Differing from other diseases, there is no pathognomonic blood test and only hematological and biochemical investigation can lead to certain metabolic illnesses and can allow the functional evaluation of the affected organs.

The most accurate diagnosis is the histopathological one, finding the type of fibril protein is essential as it will then guide towards the correct treatment.

It is useful to classify amyloidosis in 2 categories, by anatomical and clinical criteria:

• Systemic amyloidosis, where the deposits of amyloid are distributed systemically, often on the walls of blood vessels and/or conjunctive tissue

• Localized amyloidosis where the amyloid deposits affect a certain tissue or organ

The deposits of systemic amyloid rarely regress, tending to increase inexorably, leading to the distortion of the structure and altering of the function of the tissue they are localized in. This is why the systemic amyloidosis types are very severe illnesses, which lead to major morbidity. Also, because there is no real efficient treatment, the majority of cases have a bleak prognosis.

Localised amyloidosis is more frequent, usually being clinically silent and often being discovered by chance, at necropsy.

The deposits of amyloid in certain tissues are a universal illness marker. In the last two decades there is a plethora of scientific research of amyloidosis. This scientific effort has had numerous and valuable successes. The most important results of this research have been:

I. The frequency of amyloid deposits is higher than the estimated one. They are more frequent in older individuals, whilst senile amyloidosis affects over 15% of over 65s and practically 100% of over 85s. The exact role of these deposits, a cause/effect of age related pathology, is not yet determined, but it is clear that the amyloidosis is a very common illness

II. The amyloid deposits are involved in the anatomic pathology of dementia and transmittable spongiform encephalopathy. Dementia, defined as the global decline of cognitive functions, has reached an alarming frequency, especially due to increased longevity.

The study of transmittable spongiform encephalopathy has lead to the discovery of some infectious agents consisting exclusively of protein matter. This discovery represents one of the most important microbiologic scientific achievements of the century

III. It has been proven that the acute phase reaction, as a general defence mechanism of the body against aggression, is involved in the amyloidosis pathogenesis.

Despite significant progress, there are still a lot of unknown issues which make amyloidosis one of the most frustrating internal medicine domains. 

In certain types of amyloidosis, research over a long period of time has proved that therapy can reduce the amount of precursory protein amyloid fibrils, leading to the preservation of the affected organ’s function and prolonging life.

Despite the optimism given by this strategy, we have to recognize that in the case of a large type of amyloidosis, Alzheimer or systemic types for example, any therapy attempted so far has failed. This leads to continuous research in order to find a new way if inhibiting it, stopping the deposits and help reduce amyloid levels.

Often the frequency of amyloidosis is underestimated, being involved in the pathogenesis of various illnesses with apparently obscure aetiology.

Deposits of amyloid in the brain and cerebral blood vessels are the key histopathological markers in Alzheimer’s, whilst there is amyloid at Langerhans island level in DZ type II. Amyloid deposits in bones, joints and periarticular structures also affect numerous patients on haemodialysis, leading to a serious level of morbidity.

Finally, there are 2 particular types of amyloidosis: systemic AL amyloidosis (also known as primary amyloidosis, affecting multiple myeloma and other B-cellular dyscrasias) and secondary AA amyloidosis, caused by chronic infections and inflammatory diseases.

Hereditary amyloidosis is a rare illness, typical to some geographic areas, and often manifests itself as familial amyloid polyneuropathies.

**Amyloidosis pathogenesis**

The intimate mechanisms of the formation, deposit and persistence of amyloid fibrils are still uncertain. However, regardless of the remarkable aetiological and biochemical diversity of amyloidosis, the majority of cases feature a few common factors which interact during the course of amyloidosis:

**I. Increased production of precursor amyloid fibrils** is the most important mechanism, its structure usually abnormal (secondary structure with β linear pleated sheet configuration and quaternary structure resistant to enzyme digestion). Fifteen different proteins with low molecular weight have been proved to form amyloid fibrils.

**II. Increased precursor serum level through:**

• Overproduction

• Altered clearance

• Both.

**III. Abnormal processing (promoter mechanism)** verified by the fact that only a small amount of patients which fulfil the first 2 conditions, actually develop amyloidosis. Therefore, there is presumably an incomplete proteolytic degradation (not enough to eliminate the precursor completely)

• Altering of the proteolytic physiological activity

• Antiprotease balance against protease

**IV. Additional promoter mechanisms**

Due to variable speeds of amyloid deposits, a hypothesis was formulated concerning the existence of extra local mechanisms (at the source of amyloid deposits) or systemic, which determine the tissue deposit pattern. However it is possible that their build-up in deposits at the same time be a post fibril formation event. There are numerous potential additional promoters, none of which have been experimentally proven yet. For example some enzymes and/or unidentified tissue enzyme inhibitor, amyloid P component, GAG, anorganic compunds as well as AEF (amyloid enhancing factor), have been obtained form the spleen of certain animals which had amyloid introduced experimentally. This pathogenetic model has various unknowns but it is still useful as conceptual support.

**Amyloidosis reversibility**

In mice and rabbits, experimental amyloid deposits decrease slowly then disappear a few weeks after stopping the amylogenetic stimuli. There is a noticeably greater mobility of spleen and hepatic deposits compared to renal ones, its presence in the kidneys being caused by a weak representation of the phagocytic system at this level.

Humans do not fulfil the same conditions: the persistence of amyloid deposits is the rule, whilst their mobility and disintegration through the most aggressive and sophisticated methods, is the exception.

A significant experiment on humans has been carried out by Sagher. The subject suffered from lichenous amyloidosis localized in his legs. The author applied crossed grafts using teguments from his arms. Sagher then analyzed the evolution of the grafts over a few years, through a series of biopsies and hypodermic shots with Congo Red. Normal tissue grafted to the area of the leg fully affected by amyloidosis, amyloid deposits appeared after 5 years. The regression of amyloid in the leg tissue, grafted to the healthy arm, started after 8 years and was complete after 13 years, when the reaction to Congo Red came out negative.

**Amyloidosis diagnosis**

Amyloidosis diagnosis goes through 3 phases: suspicion, confirmation, statement.

Suspicion of amyloidosis should be compulsory to the physician, in one of the following circumstances:

1. chronic disease (over 10 years) proved to cause amyloidosis (poliarthritis, rheumatism, tuberculosis, broncho-pulmonary discharge, multiple myeloma, osteomyelitis), especially when associated with:

a. hepatomegaly

b. splenomegaly

c. cardiac disease with no other apparent cause

d. Malabsorption syndrome

e. Nephromegaly with proteinuria to nephrotic syndrome

2. Autosomal dominant heredo-familial syndromes characterised by peripheric neuropathy, nephropathy, cardiomiopathy

3. Patients with untreatable,congestive cardiac failure of unknown cause and with normal heart size

4. Patients with nephrotic syndrome of unknown cause

5. Patients with multisystemic non-inflammatory disease which affects the heart, blood vessels, muscles, digestive system, kidneys, spleen

6. Other: unexplained polyneuropathies, carpal tunnel syndrome, stage I multiple myeloma ( in the absence of anemia, hypercalcemia, lytic osteopathy).

Confirmation of amyloidosis is done through biopsy techniques. The biopsy is usually undertaken on the affected tissue. However, as shown above, positive tests done on other tissue areas are also representative. Unfortunately, biopsies only extract small tissue samples which carry limited information regarding the distribution, spread and natural history of amyloid deposits. The “gold standard” still remains the red-green birefringence of samples when examined with intense polarized light after Congo red colouring. The immunohistochemical colouring is the most direct method for identifying amyloid fibril types; however, it is often technically difficult and doesn’t offer clear results.

**Fig. 1:**
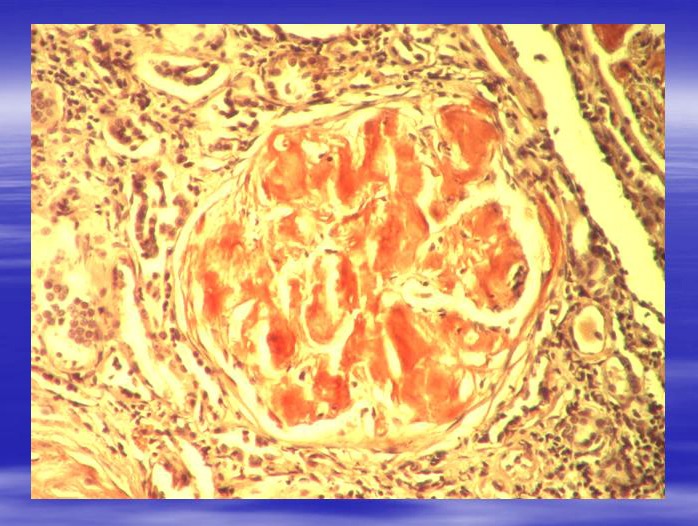
Glomerulus with big amyloid deposits (1) located in the mesangial area and in glomerular capillary walls. Toluidine blue staining. Ob.x20. Renal Morphopathology Laboratory of C. Davila Hospital.

**Fig. 2:**
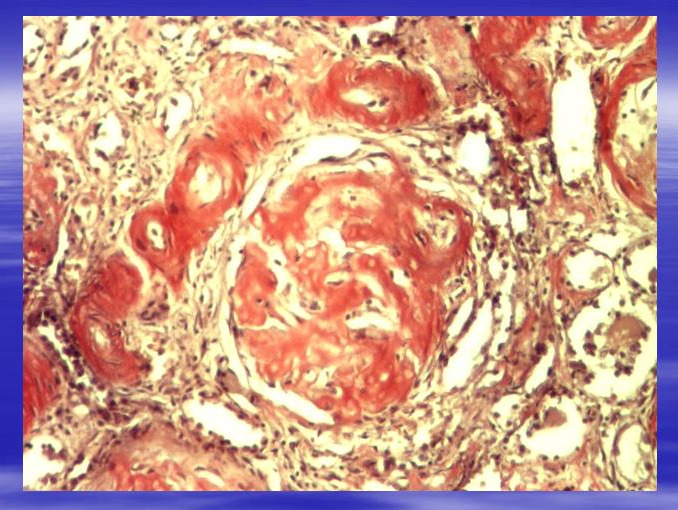
Glomerulus with multiple deposits of amyloid. Congo red staining. Ob.x20. Morphopathology Laboratory of C. Davila Hospital.

**Fig. 3:**
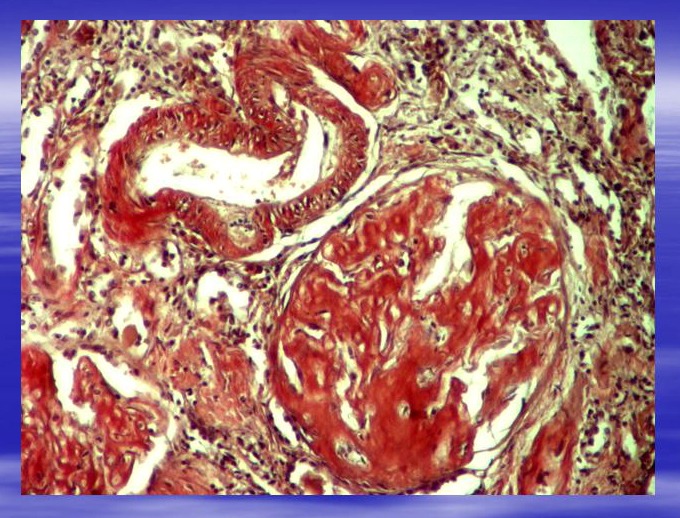
Glomerulus (G) and artery (A) with multiple deposits of amyloid. Congo red staining. Ob.x20. Morphopathology Laboratory of C. Davila Hospital.

**Fig. 4:**
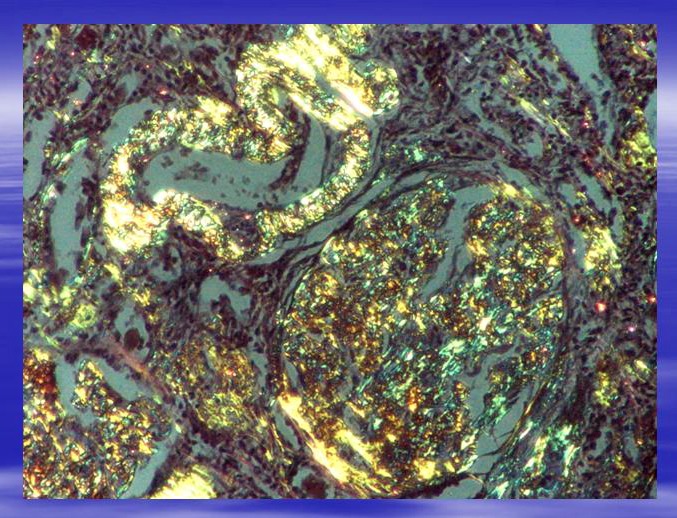
Same area as in Fig.3 seen in polarized light. Characteristical yellow-green birefringence. Ob.x20. Morphopathology Laboratory of C. Davila Hospital.

**Fig. 5:**
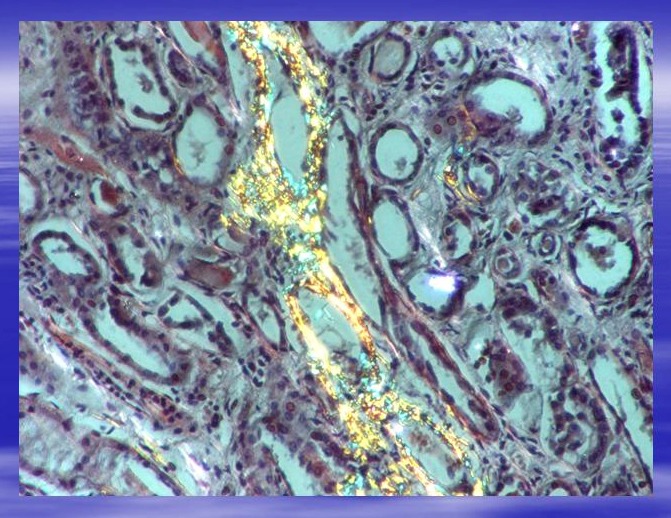
Tubules with parietal deposits of amyloid. Polarized light. Ob.x20. Morphopathology Laboratory of C. Davila Hospital.

**Amyloidosis prognosis**

Systemic amyloidosis is a very serious illness. Its vital prognosis is only 1-4 years. The main causes of death are renal failure, cardiac arrhythmia leading to sudden death, untreatable cardiac failure and gastrointestinal hemorrhage.

In comparison, AL amyloidosis prognosis is far worse than AA. Following an experiment undertaken within Mayo Clinic (*Kyle, Bayrd, 1975; Kyle, Greipp, 1983; Kyle* şi colab. 1986) on over 400 AL amyloidosis cases, the average survival period has been of only 12-15 months, and even lower when associated with multiple myeloma, cardiac failure (51%), renal (15%) or hepatic failure and infections (15%). These have been the major causes of death.

This somber perspective of all patients affected by amyloidosis constitutes a strong motivation for further scientific research meant to explain the pathological and complex mechanism of the disease and to create efficient therapies. We believe that discovering the mysteries of amyloidosis pathogenesis is the key to other unsolved degenerative or malign conditions.
